# Strategies, facilitators and barriers to implementation of evidence-based practice in community nursing: a systematic mixed-studies review and qualitative synthesis

**DOI:** 10.1017/S1463423618000488

**Published:** 2018-08-02

**Authors:** Amy Mathieson, Gunn Grande, Karen Luker

**Affiliations:** 1 Division of Nursing, Midwifery and Social Work, The University of Manchester, Manchester, UK; 2 Professor of Palliative Care, Division of Nursing, Midwifery and Social Work, The University of Manchester, Manchester, UK; 3 QNI Professor of Community Nursing and Deputy Director of NIHR CLAHRC for Greater Manchester, Division of Nursing, Midwifery and Social Work, The University of Manchester, Manchester, UK

**Keywords:** community nursing, community/home care, evidence-based practice, implementation, systematic review

## Abstract

**Aim:**

To appraise and synthesize empirical literature on implementation of evidence within community nursing. To explore the use of implementation theory and identify the strategies required for, and the barriers and facilitators to, successful implementation within this context.

**Background:**

There is an international consensus that evidence-based practice can improve outcomes for people using health and social care services. However, these practices are not always translated into care delivery. Community nursing is a relatively understudied area; little is known about how innovations in practice are implemented within this setting.

**Methods:**

Systematic mixed-studies review, synthesizing quantitative and qualitative research. The electronic databases AMED, PsycINFO, Ovid Medline, CINAHL Plus, ASSIA, British Nursing Index and EMBASE were used. Two grey literature databases were also searched: OpenGrey and EThOS. English language, peer-reviewed papers published between January 2010 and July 2017 were considered. Criteria included implementation of an innovation and change to practice within adult community nursing. An approach called Critical Interpretive Synthesis was used to integrate the evidence from across the studies into a comprehensible theoretical framework.

**Results:**

In total, 22 papers were reviewed. Few studies discussed the use of theory when planning, guiding and evaluating the implementation of the innovation (*n*=6). A number of implementation strategies, facilitators and barriers were identified across the included studies, highlighting the interplay of both service context and individual factors in successful implementation.

**Conclusion:**

Implementation is an expanding area of research; yet is challenged by a lack of consistency in terminology and limited use of theory. Implementation within community nursing is a complex process, requiring both individual and organizational adoption, and managerial support. Successful adoption of evidence-based practice however, is only possible if community nurses themselves deem it useful and there is evidence that it could have a positive impact on the patient and/or their primary carer.

## Introduction

For decades, evidence-based practice (EBP) has been an aspiration for health service providers. The World Health Organization (WHO) has stated that healthcare provision should be based on the best available evidence. Healthcare professionals are expected to engage with evidence and practice in line with it. Professional regulatory bodies such as the Nursing and Midwifery Council include the expectation that nurses deliver EBP in all settings (Brooke and Mallion, 2016; Mallion and Brooke, 2016). However, gaps still exist between research evidence, changes to practice and improved outcomes for patients (Wilson *et al*., 2010; Grimshaw *et al*., 2012).

Previous research, exploring nurses’ beliefs, skills and knowledge of EBP, has reported that nurses encounter various barriers to EBP implementation, resulting in a lack of engagement. Barriers frequently reported include lack of time, staff shortage, heavy patient caseload, family commitments, limited knowledge of EBP and negative beliefs toward it, and limited academic skills (Mallion and Brooke, 2016). Many publications however, fail to address why and how implementation processes have worked, or why attempts failed. This lack of concern with the process of change is another barrier to the application of best evidence in practice (Bryar and Bannigan, 2003). There have been calls by policy makers and researchers to use theory to explore these processes (Mcevoy *et al*., 2014).

The majority of nursing implementation science research has been conducted in acute hospital settings (Estabrooks *et al*., 2002; Lucero *et al*., 2009; Van Bogaert *et al*., 2009; Brooke and Mallion, 2016). Although care in the community has expanded globally, literature exploring implementation of EBP in this setting remains limited, including implementation strategies used by community nurses (Brooke and Mallion, 2016).

Lessons may be learnt from hospital and nursing home settings; however, community nursing is a unique setting that requires specific implementation strategies. Furthermore, it has been agreed that implementation studies in nursing are preoccupied with ‘education’ as a strategy including lectures and discussion sessions, feedback and reminders (van Achterberg, 2013); other potential strategies are seldom explored.

The aim of this review is to explore the use of implementation theory and identify the strategies required for, and the barriers and facilitators to, successful implementation within community nursing. The purpose is to add to the knowledge on effective implementation within this context.

In this paper, we use the term ‘implementation’ and ‘community nursing’. Internationally, community nursing is often referred to as ‘home care nursing’. In the United Kingdom, Australia and Sweden these nurses are referred to as ‘district nurses’. We define community nursing as the work of caring for people in their homes, rather than an institution. We define implementation as ‘[The] active and planned efforts to mainstream an innovation within an organization’ (Greenhalgh *et al*., 2004, p. 582). While dissemination is an important component in implementation science, this review is focused on the implementation only of innovations designed to enhance care.

## Method

As the topic area of this review is context-sensitive, a design that provides a practical understanding of the phenomenon is required. The approach taken was therefore a systematic mixed-studies review and convergent qualitative synthesis (Pluye and Hong, 2014). To maximize transparency, where appropriate, we have reported our review in line with the PRISMA statement (Moher *et al*., 2009).

The standard systematic review steps were taken, whereby the reviewer (A.M.) identified, selected, appraised and synthesized qualitative, quantitative and mixed-methods studies; with all papers reviewed independently by the three authors. Authors discussed disagreements and a consensus was met. For pragmatic reasons, a time limit for the literature was imposed. To ensure all relevant literature within this time limit was captured, a number of databases were searched from different fields.

## Search strategy

**Figure 1:**
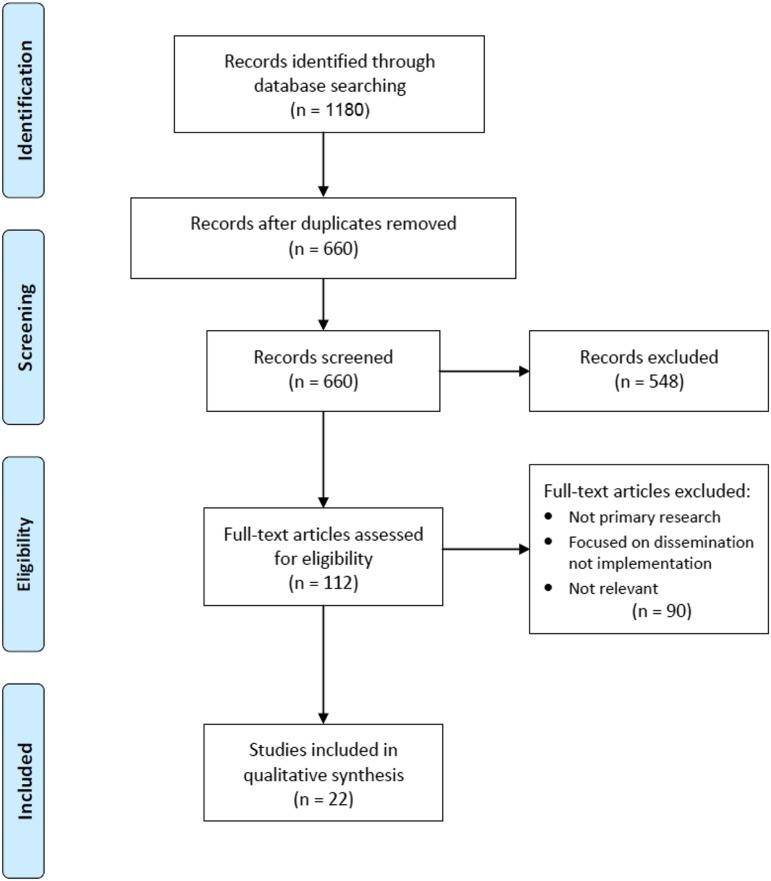
PRISMA flowchart of search strategy

The search strategy (Figure 1) included papers published between January 2010 and July 2017 identified from the electronic databases AMED, PsycINFO, Ovid Medline, CINAHL Plus, ASSIA, British Nursing Index and EMBASE. The grey literature databases OpenGrey and EThOS were also searched. A Boolean search was conducted using the following terms ‘Implement*’; ‘Evidence-based Medicine’; ‘Evidence-based Practice’; ‘District Nurs*’; ‘Community Health Nurs*’; ‘Home Care Nurs*’ (Table 1). The search strategy identified a total of 660 titles. Retrieved articles were considered for inclusion if they were peer-reviewed primary research published in English and met the inclusion criteria of implementation within community nursing. This was restricted to adult general nursing. The scale on which the innovation (EBP) was implemented was not stipulated. Implementation within a children community nursing setting, and community mental health nursing studies in the absence of physical illness, were excluded. In total, 22 papers were identified as relevant.

**Table 1:** Search key with Boolean operators

Search line	Term
1	Implement*
2	Evidence-based Practice
3	Evidence Based Practice
4	Evidence-based Medicine
5	Evidence Based Medicine
6	OR/1-5
7	District Nurs*
8	Community Health Nurs*
9	Home Care Nurs*
10	OR/7-9
11	6 AND 10

A separate search was conducted in the Cochrane Database for Systematic Reviews to explore publications on the same subject matter to avoid unnecessary duplication.

## Quality appraisal

The 22 included studies were appraised using the assessment template for disparate data developed by Hawker *et al*. (2002) (Box 1). This checklist consists of nine questions, each of which has four sub-categories, allowing for the calculation of a summed score indicating the methodological quality. Using this tool and following the appraisal prompts, each paper was rated on a scale from 36 (Good) to 4 (Very poor). The studies were independently assessed by at least two reviewers. Most studies were assessed to be of reasonable quality, with scores ranging from 20 to 33 (Table 2). Papers were not excluded based on quality assessment.

**Table 2:** Summary of the included studies

Authors and year	Country	Innovation/type of intervention	Research Design and Method	Quality Score
Amacher *et al*. (2016)	Switzerland	Falls Prevention Programme (FPP)	Mixed-methods: qualitative interviews followed by questionnaire Post-implementation	28
Andrew *et al*. (2013)	UK	The Dignity Care Pathway (DCP)	Qualitative: focus groups Pre–post-implementation	22
Annells *et al*. (2011)	Australia	Best practice mental health screening and referral clinical pathway for generalist community nursing	Mixed methods: evaluation surveys and focus groups Pre–post-implementation	26
Brennan *et al* (2010)	USA	Technology-enhanced practice (TEP): usual care supplemented with a web-based resources	Quantitative: surveys. Post-implementation	26
Byron and Hoskins (2013)	UK	Verification of expected death (VOED) Policy	Qualitative: semi-structured interviews Post-implementation	32
Doran *et al.* (2013)	Canada	A clinical information system (CIS) in a community setting	Mixed methods: interviews; focus groups; and surveys Post-implementation	25
Haycock-Stuart and Kean (2013)	UK	Policy to shift location of care from secondary, acute care settings to primary, community care settings	Qualitative: semi-structured interviews and focus groups Post-implementation	31
Joy *et al*. (2015)	UK	Foam dressing	Quantitative: reduction range of dressings used, frequency of dressing changes, visits and overall cost Pre- and post-implementation	26
Kapp (2013)	Australia	Australian Wound Management Association (AWMA) Guidelines	Quantitative: surveys and client profiles Pre–post-implementation	23
Murray *et al.* (2011)	UK	(1) Choose and Book (C&B) system in a hospital trust and the lead Primary Care Trust providing referrals to the hospital (2) Picture Archive and Communication System (PACS) in one acute hospital trust (3) Community Nursing Information System (CNIS) for district nurses (DNs)	Qualitative: semi-structured interviews Post-implementation	32
Nilsson *et al.* (2010)	Sweden	An electronic messaging program via computers and mobile phones with an internet connection	Qualitative: semi-structured interviews Pre–post-implementation	28
Nordmark *et al*. (2016)	Sweden	Discharge Planning Process (DPP) and information exchange through an electronic information system.	Qualitative: observations; interviews; registered adverse events and system failures; web-based survey Post-implementation	30
Paquay *et al.* (2010)	Belgium	The Belgian Guidelines for Prevention of Decubitus Ulcers (BGPDU)	Quantitative: case report forms Pre–post-implementation	32
Pare *et al.* (2011)	Canada	SyMO – software to plan and organize nursing activities in patients’ homes	Mixed-methods: structured questionnaire, semi-structured interviews were conducted with nurses, and a postal questionnaire was sent to patients. Pre–post-implementation	28
Sherman *et al.* (2016)	UK	Case management within community nursing practice.	Qualitative: focus groups. Pre-implementation	33
Smith *et al*. (2013)	Sweden	Preventive Home Visits (PHV)	Quantitative: cluster-controlled trial Post-implementation	26
Tapper *et al.* (2012)	Canada	Tablet computers.	Quantitative: online surveys Post-implementation/evaluation	20
Taylor *et al*. (2015)	UK	Telehealth to monitor patients with COPD and Chronic Heart Failure	Qualitative: semi-structured interviews Post-implementation	31
Vabo *et al*. (2016)	Norway	Guideline for assessing individual needs and instructions for using the guideline, including nursing documentation routines and training; in the context of move to a common electronic health record (improved content and quality of nursing documentation)	Qualitative: focus groups and interviews Post-implementation	31
Van der Plas *et al.* (2014)	The Netherlands	PaTz (Palliative Thuis Zorg – Palliative Care at Home)	Mixed methods: questionnaires and focus groups Post-implementation	32
Whittemore *et al.* (2013)	USA	A modified diabetes prevention program (mDPP) provided by homecare nurses to adults of public housing communities at-risk for type 2 diabetes	Mixed methods: secondary analysis of a cluster randomization pilot study; interviews Post-implementation	32
Wilcox *et al.* (2010)	USA	The Heart Healthy and Ethnically Relevant (HHER) Lifestyle Program	Quantitative: provider/nurse and client encounters were audio-recorded Pre-implementation	29

Box 1 Assessment form (Hawker *et al*., 2002)

**Table:** 

Author and Title:					
Date:					
	Good	Fair	Poor	Very poor	Comment
1. Abstract and title					
2. Introduction and aims					
3. Method and data					
4. Sampling					
5. Data analysis					
6. Ethics and bias					
7. Findings and results					
8. Transferability/generalizability					
9. Implications and usefulness					
Total

1. Abstract and title: Did they provide a clear description of the study?

2. Introduction and aims: Was there a good background and clear statement of the aims of the research?

3. Method and data: Is the method appropriate and clearly explained?

4. Sampling: Was the sampling strategy appropriate to address the aims?

5. Data analysis: Was the description of the data analysis sufficiently rigorous?

6. Ethics: Have ethical issues been addressed, and what has necessary ethical approvals gained?

7. Results: Is there a clear statement of findings?

8. Generalizability: Are the findings of this study transferable (generalizable) to the wider population?

9. Implications and usefulness: How important are these findings to policy and practice?

## Data abstraction and synthesis

Key areas of each of the included papers were extracted by A.M. including: research design and methods; innovation/type of intervention; use of theory; participants and setting; outcome measures; findings; implementation data; barriers/challenges; and facilitators. The three authors discussed the data extracted for all papers. All the extracted data were analyzed using the same synthesis method.

Since the included studies were heterogeneous regarding design and outcome, we used an interpretive rather an aggregate approach to synthesize the evidence, namely Critical Interpretive Synthesis (CIS) (Dixon-Woods *et al*., 2006). This approach was adopted to explore the factors shaping implementation within community nursing, and aimed to produce an empirically grounded framework, which can be used in practice (Dixon-Woods *et al*., 2006). Previous reviews that have used CIS have revealed the appropriateness of this approach for answering questions concerning the influence of context on effectiveness (Flemming, 2010a; 2010b); in this instance, the impact of context on the success or not of implementation, and effectiveness of certain strategies and facilitators.

The extracted data were initially analyzed thematically. Recurring themes were identified by examining the evidence, and concepts (implementation strategies, barriers and facilitators) were generated. Concepts were refined by comparing the framework to the data and exploring relationships between them (Dixon-Woods *et al*., 2006). To produce a synthesizing argument, the concepts across the studies were then critically examined in terms of similarities (reciprocal transition) and differences (refutational translation) (Pluye and Hong, 2014). Similar concepts were translated into each other, and contradictions between studies were explored, until the most adequate concepts to explain the phenomena were chosen (Dixon-Woods *et al*., 2006). This allowed a ‘line of argument’ to be built, which integrated the evidence across the studies into a framework comprising the most prominent implementation strategies, barriers and facilitators constructs, and relationships between them.

## Results

Nine qualitative papers, seven quantitative and six mixed-method studies were identified (Table 2). ‘Post-implementation’ studies (*n*=13) evaluated implementation efforts and impact of the innovation. ‘Pre–post-implementation’ studies (*n*=7) planned implementation, implemented the innovation and evaluated efforts. The remaining two studies were pre-implementation. The majority of post-implementation papers reported on large-scale studies evaluating a change led by an organization. Many of these reported *post hoc* implementation strategies or organizational support strategies. The included pre–post-implementation studies were mostly collaborative research projects, whereby the research team worked with potential adopters at an organizational or community level. Implementation strategies reported in these papers were mostly researcher-led. Similarly, the two pre-implementation papers reported on researcher-led projects.

Only six of the studies discussed the use of theory when planning, guiding and evaluating the implementation. Tapper *et al*. (2012) and Andrew *et al*. (2013) both used theory when planning implementation. Tapper *et al*. (2012) used Powell-Cope *et al*.’s (2008) conceptual framework on patient care technology when selecting the innovation, considering factors that may impact implementation, and developing the questions for the online survey. Andrew *et al*. (2013) used the integrated knowledge transfer approach described by Graham (2007) to guide the collaborative research project to implement a palliative care intervention.

Murray *et al*. (2011), van der Plas *et al*. (2014), Nordmark *et al*. (2016), and Vabo *et al*. (2016) applied theory to the findings to explore factors that impacted implementation. For Murray *et al*. (2011) and Nordmark *et al*. (2016) this was Normalization Process Theory; used as a framework to analyze qualitative findings. Similarly, Vabo *et al*. (2016) discussed the study’s findings using the PARiHS framework. van der Plas *et al*. (2014) applied Petrie’s framework for inter-professional work to the findings (Petrie, 1976).

**Figure 2:**
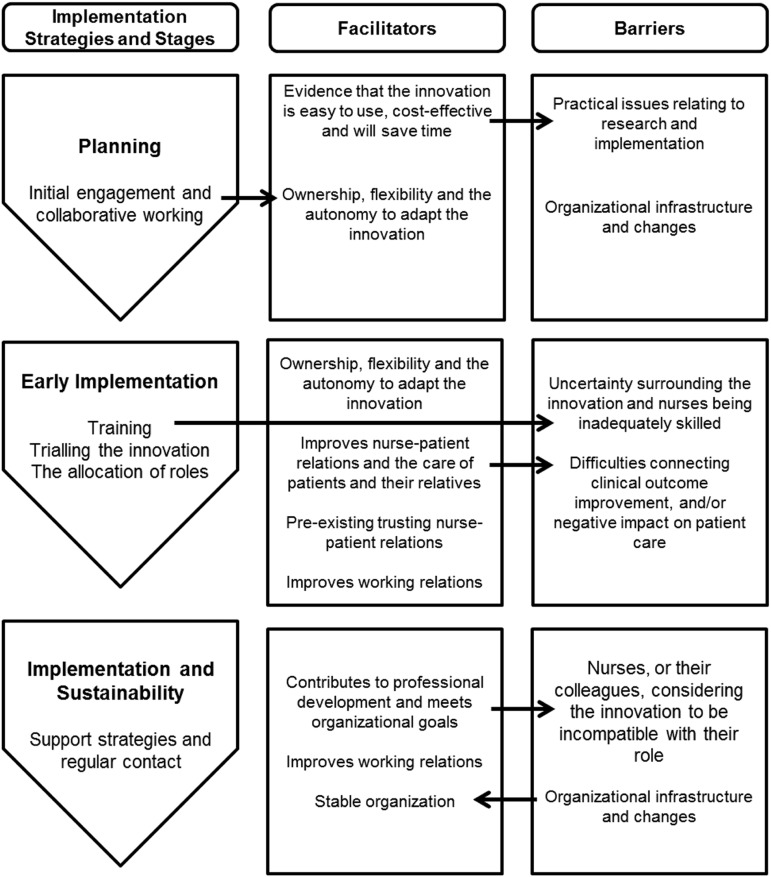
Relationship between implementation strategies, facilitators and barriers

In addition to the general observations, implementation strategies, facilitators and barriers were identified (see online Supplementary File 1 for a summary) and synthesized. Implementation strategies are defined as techniques used for the adoption and sustainability of an innovation (Proctor *et al*., 2013), often before implementation. Facilitators ease the implementation process once underway and refer to support that occurs within the context (Stetler *et al*., 2006) including resources, culture and values (Kitson *et al*., 2008); and barriers inhibit. The reported barriers, with the exception of ‘organizational infrastructure and changes’, are the antithesis of the identified facilitators. The barriers and facilitators are therefore discussed in parallel. Figure 2 illustrates how the strategies, facilitators and barriers inter-link. The following sections present the result of the synthesis.

## Implementation strategies

### Training and support strategies

Training nurses on how to use the innovation before it is implemented contributed to successful adoption. Most of the studies discussed the importance of the nurses’ confidence in using the innovation, with inadequate training often stated as a barrier. This was particularly evident when the innovation was technological. Length of training across the studies varies. Joy *et al*. (2015) provided a two week training and education phase when exploring the implementation of a new wound dressing. In contrast, a 3 h course was offered to healthcare professionals in Vabo *et al*.’ study (2016) and one day course for the District Nurses in Sherman *et al*.’ project (2016). This one day course was later extended to two days, and was followed by a group discussion four weeks after, as implementation was unsuccessful. Evidence that the wound dressing was successfully adopted (Joy *et al*., 2015) suggests a long training period is a contributing factor to successful adoption. In addition, continued support after initial training, encouraged adoption (Annells *et al*., 2011; Tapper *et al*., 2012; Kapp, 2013). Continued support included support for staff from other staff members; ‘safe spaces’ for staff to share positive and negative experiences of using the innovation; telephone or face-to-face contact with the researchers; and management support and allocation of organizational resources. For example, nurses in Kapp’s study (2013) were provided with telephone and email support throughout implementation. Practice guidelines were successfully implemented and pressure risk screening became a well-adopted practice (Kapp, 2013).

### The allocation of roles

A strategy used in seven of the studies was the allocation of roles, such as an implementation team, key implementers or individuals responsible for monitoring the implementation process. This included the appointment of nurses into employed positions (Kapp, 2013; Smith *et al*., 2013; Whittemore *et al*., 2013; Nordmark *et al*., 2016) and volunteers, whose involvement in the study was in addition to their day-to-day role (Taylor *et al*., 2015; Vabo *et al*., 2016). For instance, Nordmark *et al*. (2016) in their study exploring the implementation of discharge planning process (DPP) found that discharge planners were appointed, who had a clear role and legitimate time to perform the DPP. Discharge planners were engaged with their work task, became experts in the area and the quality of the DPP improved (Nordmark *et al*., 2016). Where voluntary ‘key nurse’ roles were used, these were able to support staff during the implementation, and connect potential adopters with the innovation and the research team to enable them to participate in the studies’ data collection (Taylor *et al*., 2015; Vabo *et al*., 2016). For example, Taylor *et al*. (2015) recruited a local lead collaborator in each of the participating sites. ‘Local champions’ also emerged once implementation had begun and facilitated implementation. Local champions were key enablers of adoption through their promotion of telehealth and the support they provided to other staff (Taylor *et al*., 2015). Support from local champions was provided after the initial training by offering information, advice and sharing positive experiences. Having a clear visible nurse leader in addition to the research team, positively contributed to implementation.

## Facilitators

### Evidence that the innovation is easy to use, cost-effective and will save time

Practical issues, such as saving clinical time, cost-effectiveness and ease of use were important considerations for community nurses when implementing an innovation. In Murray *et al*.’s (2011) study exploring the use of the Community Nursing Information System, District Nurses claimed the Personal Digital Assistant devices were cheap, robust and portable, allowing nurses to feel comfortable carrying them when visiting patients. Similarly, in Kapp’s study (2013), respondents found the electronic risk tool, part of the Australian Wound Management Association Guidelines, was easy to use, and implementers were satisfied with the length of time to complete the tool. In contrast, the nurses in Annells *et al*.’s study (2011) claimed they were very ‘time poor’ and therefore were not always able to use the ‘lengthy’ mental health screening and referral clinical pathway. Furthermore, the pathway required time to become accustomed to it, which arguably community nurses do not have. A recurring theme was the time restrictions experienced by community nurses due to the nature of their work and increased demands upon the service. This inhibited community nurses’ participation in research and their ability to implement the innovation. Thus evidence across the studies suggest if the innovation saves clinical time, nurses will be more likely to continue with its implementation and may be encouraged to use the innovation in the future. van der Plas *et al*. (2014), for example, found that the PaTz (an acronym for ‘PAlliatieve Truis Zorg’; palliative care at home) saved healthcare professionals’ time. General Practitioners and District Nurses worked as a team and time was spent more efficiently (van der Plas *et al.*, 2014).

### Ownership, flexibility and the autonomy to adapt the innovation

A ‘bottom-up’ approach, including early engagement and collaborative working, and the ability of community nurses to tailor the innovation to meet individual needs was an important facilitator (Brennan *et al*., 2010; Paquay *et al*., 2010; Andrew *et al*., 2013; Haycock-Stuart and Kean, 2013; Smith *et al*., 2013; Joy *et al*., 2015; Taylor *et al*., 2015; Vabo *et al*., 2016). This was evident in Haycock-Stuart and Kean’s (2013) study exploring the implementation of a policy to shift care from acute care settings to community care settings. They found that the current top-down approach was not successful as there was no ‘ownership’ of the policy from frontline staff. The ‘top-down approach’ was at odds with the grass root service organization and delivery in the community setting, causing resistance from staff (Haycock-Stuart and Kean, 2013). Haycock-Stuart and Kean (2013) suggests that policy implementation should be negotiated with frontline community nurses, as a ‘shared vision’ is important for successful implementation. This was evident in Andrew *et al*.’s study (2013), which explored the implementation of the Dignity Care Pathway (DCP). Focus groups were conducted before implementation to gain ‘context-specific evidence’, and to assess if the DCP ‘fitted with nurses’ practice’ (Andrew *et al*., 2013). Possibly as a result of this engagement, the authors found that the assessment tools within the DCP could be used flexibly to accommodate patients’ individual needs and was applicable in everyday practice. Furthermore, working closely with the research team supported a collaborative relationship with the implementers, which facilitated the sharing and solving of issues during the study.

### Improves nurse–patient relations and the care of patients and their relatives

Evidence that the innovation improves nurse–patient relations and patient care was a contributing factor in community nurses’ decision to adopt it. This was a facilitator for 14 of the 22 studies. For example, Nilsson *et al*. (2010) found that District Nurses initially expressed concern with using the information and communication technology (ICT), and thought it may be difficult to manage if many patients used the technology at the same time. Once accustomed to the use of ICT, the District Nurses said ‘a routine’ using the messaging program helped them to organize their work and was ‘what the ill person needed’, positively contributing to their relationship with the patient (Nilsson *et al*., 2010). In contrast, in Vabo *et al*.’s (2016) action research project implementing improved nursing documentation, nurses felt the system forced them to focus on isolated aspects of care. This was incompatible with the healthcare professionals’ values and goals in providing holistic care. Nurses therefore reverted back to ‘old habits’, stating their preference to spending time with the patient instead of documenting (Vabo *et al*., 2016).

### Pre-existing trusting nurse–patient relations

There is evidence that nurses’ experience and rapport with patients assisted with the successful implementation of an innovation. Annells *et al*. (2011) found that rapport and trust between nurses and clients contributed to the pathway use. The pathway was successfully implemented even though many of the nurses believed they were inadequately skilled to undertake the generalist nursing care of people with mental health problems (Annells *et al*., 2011). This could be attributed to the trusting relationships between the nurses and clients, which facilitated implementation and eased potential barriers. Similarly, Andrews *et al*. (2013) found that the experienced nurses’ main criteria, when deciding to introduce the Dignity Care Pathway to patients, included having an established rapport, knowing the patients’ understanding of their prognosis, and consideration of family dynamics. Experienced nurses were also able to decide when to introduce the pathway; implementation may have been difficult for less experienced nurses. This finding however, revealed that the innovation was only being used with a select group of patients. The innovation had not been mainstreamed within the organization, and instead highlights adoption on an individual level.

### Contributes to professional development and meets organizational goals

Nine of the studies claimed that professional development associated with using the innovation – maintaining existing skill-sets, developing new skills and knowledge – and the innovation serving to meet organizational goals, encouraged community nurses to adopt it. In turn, the organization offered appropriate support to assist with its successful implementation. Doran *et al*. (2013) found that access to electronic resources supported nurses’ learning and enhanced knowledge; these were found to be associated with nurses’ willingness to use the BlackBerry to document patient outcomes through the CIS. Furthermore, positive attitude towards both the employer and their values increased nurses’ willingness to adopt CIS (Doran *et al*., 2013). The opportunity to learn, have access to resources and develop new skills appeals to community nurses, and may encourage them to implement an innovation if it supports them to do so. Byron and Hoskins (2013), when exploring the implementation of the ‘verification of expected death’ (VOED) policy, found that over half the nurses agreed that regular updates on VOED would help maintain skills and competency. Junior staff would also have the opportunity to learn from more experienced nurses. As a result, VOED had become part of an induction programme (Byron and Hoskins, 2013).

### Improves working relations

For Murray *et al.* (2011), Nordmark *et al.* (2016), Smith *et al*. (2013), van der Plas *et al.* (2014) and Whittemore *et al.* (2013) improved working relations had a positive impact upon the implementation of the respective innovations. Nordmark *et al*. (2016) found that hospital, primary healthcare and community care providers meeting together at the Discharge Planning Conference (DCP) overcame organizational boundaries and discussing the DPP facilitated a collective view of the process. Similarly, van der Plas *et al*. (2014) found that General Practitioners and District Nurses worked well together when they met six weekly. During these meetings, practitioners saw each other as more equal partners and appreciated each other’s expertise (van der Plas *et al.*, 2014). Both the innovation, and the process of implementation, improved working relations, which in turn facilitated the continued use of the innovation.

## Barrier

### Organizational infrastructure and changes

Organizational changes – restructuring and the decentralization of services – had a negative impact upon implementation. Murray *et al*. (2011) found that organizational change absorbed staff time and energy, distracting them from the e-health implementation. Possibly related to this organizational change was a perceived problem with leadership, including the disbanding of the dedicated implementation group after the first year and inadequate allocation of resources for training and support (Murray *et al*., 2011). Similarly, Taylor *et al*. (2015) found that the restructuring of community nursing teams, the integration of health and social care, and the creation of the new Clinical Commissioning Groups were all raised as barriers to the adoption of the new telehealth technologies. Staff described these changes as ‘unprecedented’ and ‘overwhelming’ (Taylor *et al*., 2015). As a result, adjustment to these changes was considered the priority. Similarly, Brennan *et al*. (2010), when exploring the implementation of technology-enhanced practice, found that the intervention was affected by infrastructure changes. A stable organization, as opposed to re-organization, therefore supports implementation within community nursing.

## Discussion

### General summary

The review has illustrated that community nurses’ decision to adopt an innovation appears to be motivated by a return on investment, including improved nurse–patient relations or patient care, contribution to professional development, or improved relations with other healthcare professionals. In addition, flexibility and the autonomy to adapt the innovation encouraged community nurses to adopt it. This mirrors community nurses’ working practices and satisfaction derived from organizing their own workload. Organizational infrastructure and change was an important barrier, with community nurses claiming they were preoccupied with, and prioritized, these changes.

Quality of the included studies varied. Across the qualitative studies, sample sizes are small, limiting generalizability. Authors of qualitative studies often compensated for the lack of external validity by adopting strategies to increase trustworthiness, such as member checking and triangulation. However, findings from the included qualitative studies in this review have been corroborated with results from the quantitative papers. Overall the quality of the included quantitative studies was higher than the qualitative or mixed-methods studies. Due to the nature of the research design, sample sizes are larger and there is greater external validity amongst the quantitative studies. For example, Sherman *et al*.’s (2016) study scored highly on Hawker *et al.*’s (2002) appraisal tool. Sherman *et al*. (2016) along with Annells *et al.* (2011) and Doran *et al.* (2013) all used validated and tested tools. In contrast, the surveys and tools used by Paquay *et al.* (2010), Tapper *et al.* (2012) and Kapp (2013) were untested and not validated.

Variation in the quality of studies, designs and samples implies implementation science in community nursing requires development. Authors using different terms to describe the same phenomena cause confusion. Implementation strategies in the literature are seldom labelled and researchers do not provide rationales for the strategies used. This review attempts to provide some harmony by presenting an overview and synthesis of strategies, barriers and facilitators.

### ‘Line of argument’: the complexity of implementation within community nursing

The identified barriers and facilitators paint a complex picture of implementation within community nursing and it does not appear to be a simple recipe of ‘do and don’t’. It is clear from the included studies, and may even be assumed, that community nursing is patient-focussed. Community nurses were therefore less likely to adopt the innovation if, by integrating it into their routine practice, existing nurse–client relations could be negatively affected. In addition, the trend across the quantitative studies to include patients as participants suggests that end-user adoption may influence implementation. Patients’ unwillingness to comply with EBP may discourage practitioners (Logan and Graham, 1998). This finding may be a result of selection bias, as papers were included if they reported on the implementation of an innovation designed to enhance care. If the innovation did not enhance care, implementation may be considered a ‘failure’. Yet our analysis also suggests that community nursing practice is driven by other factors, which may promote or inhibit implementation. These include meeting organizational goals and targets, by which community nurses’ practice is ultimately assessed; and professional development. A ‘good idea’ or evidence that the innovation improves care is therefore not enough and other conditions or facilitators are required. This underlines the importance of working in partnership with practitioners to identify research questions and develop interventions.

Training and an on-going education phase on how to use and integrate the innovation was a key facilitator found across the included papers. However, embedding this training through experience is challenging (Taylor *et al.*, 2015). Continued support is needed, which can be difficult when managing a busy caseload. A lack of time is a widely reported barrier to engagement with, and implementation of, EBP. One solution may be to hold training outside of working hours, if attendance contributes to nurses’ revalidation (Johnston *et al*., 2016). Another is support from ‘local champions’ (Taylor *et al*., 2015). Taylor *et al.* (2015) found that local champions and nurses with substantial experiential knowledge facilitated shared learning and were able to troubleshoot issues with the adoption of the telehealth. Although researchers are able to offer support, clinical buy-in and drive from the nurses who have, or wish to, adopt the innovation, is required.

Giving nurses the flexibility to use and adapt the innovation, and the need for managers to be on board to allow the time and investment of resources to implement it, were both facilitators. Haycock-Stuart and Kean’s (2013) findings suggests there is a complex interplay between the values nurses place upon an innovation, their current skill set and preparation for integrating the innovation, both from frontline staff and managers. However, findings collectively from the included studies suggest that nurses make decisions as individuals. Context clearly plays a key role here. While community nurses deliver care, they also organize it and are often responsible for their caseload (Sales, 2013). Implementing an innovation is not an exception to this; nurses are an active component within the process, and it is arguably through the process of reflection and critical thinking that change occurs (Griffiths, 2003). As Doran *et al*. (2013) highlight, community nursing is independent in nature. As most of the work community nurses do is in patients’ homes and is not supervised by managers (Walshe and Luker, 2010), community nurses can organize their own workload and dedicate time to certain tasks. This could contribute to successful implementation or not, as community nurses have a degree of autonomy and can therefore decide to adopt an innovation. This may mean adoption in community nursing is rather individualistic and therefore haphazard.

Community nurses’ work however, is not conducted in isolation. Many of the studies included other healthcare settings or participants in their analysis, such as General Practitioners; secondary care settings; and manager (Annells *et al*., 2011; Murray *et al*., 2011; van der Plas *et al.*, 2014; Taylor *et al*., 2015; Amacher *et al*., 2016; Vabo *et al*., 2016). This suggests community nurses’ interactions with other healthcare professionals, and their attitudes towards nurses’ changing practices, could act as a barrier or facilitator. Along with improvements to patient care and saving time, opportunities to work with other healthcare professionals and feeling a valued member of the team may be an acceptable ‘return on investment’ and encourage community nurses to adopt an innovation. There is also evidence that the wider organizational context can facilitate or inhibit the adoption of EBP. Working in a constantly changing environment makes mainstreaming an innovation difficult. As Taylor *et al.* (2015) found, other changes may take priority and render the introduction of another change impossible; highlighting the importance of timing when implementing an innovation. Furthermore, evidence from the included papers suggested innovations that required the re-organization of teams also resulted in resistance from adopters, which in turn created a barrier to implementation. The included papers, and literature elsewhere (Harrod *et al*., 2016; Johnston *et al*., 2016), revealed that others working in multi-disciplinary teams could act as a barrier. In order to implement an innovation within a multi-disciplinary team, adopters need to understand each other’s roles and workload (Harrod *et al*., 2016; Nordmark *et al*., 2016).

As discussed, adoption of an innovation is reliant on the decision of the community nurse. There is little evidence, however, to suggest that nurses introduce innovations themselves after engaging with EBP. This may be a result of a lack of availability of relevant research in primary care, lack of skills to appraise evidence, or the lack of awareness of change mechanisms (Bryar and Bannigan, 2003). In addition, the reactive nature of community nursing makes it difficult for nurses to plan and introduce changes, with practitioner making snap decisions in patients’ homes (Closs, 2003). Furthermore, in practice, implementation is trial and error and nurses may not have the confidence to trial the innovation. Ultimately, however, community nurses need support from the organization and/or senior colleagues. Managers are more likely to grant time, and support implementation efforts, if the innovation meets organizational goals, for example national targets, and if it is compatible with their values. This illustrates that evidence-based nursing not only depends on the availability of evidence, patient preferences and the clinical expertise of the nurse, but also the organizational resources available (Closs, 2003). This suggests that community nurses have more freedom to decide if they wish to engage with top-down implementation than to introduce an innovation without management instructions. Future implementation within the context of community nursing therefore requires a facilitated approach, acknowledging both top-down and bottom-up techniques.

### Gaps in the literature

Continued support, including collaborative working and the use local champions, were identified to be both implementation strategies and facilitators, promoting the adoption of an innovation. The other identified implementation strategies and facilitators require further testing. Future research could evaluate how these facilitators could be used to effectively overcome barriers.

There is a lack of research based on rigorous conceptual frameworks. Theory is seldom used when implementing an innovation. There are a plethora of frameworks and theories relevant to implementation research that can be used to guide implementation processes and consider sustainability (Tabak *et al*., 2012; Wilson *et al*., 2017). There is a need to test theories in a community nursing context. Furthermore, more effort should be made to understand how sustainability of implementation can be achieved (van Achterberg, 2013). As a result of limited funding, follow-up periods after the introduction of new EBP are often short and implementation science researchers are required to work within the resources available to them. We therefore suggest that future research be undertaken around the continued adoption of EBP within community nursing; and the use of identified strategies to sustain a change of practice within this context.

### Limitations

There are a number of limitations to this review, mainly relating to scope. Only English Language papers were reviewed and the quality of the papers varied. The included papers come from a wide range of countries with differing healthcare systems. However, by adopting a Critical Interpretive Synthesis approach we have attempted to be critical and clarify effective implementation strategies in a diverse and confused field. Due to inconsistency in reporting, labelling and defining these strategies have relied upon our interpretations and those of the authors’ of the included studies. In particular, the *post hoc* implementation strategies offered in the included studies are attempts by the authors to explain what did or did not work. There may be alternative explanations, and more appropriate names. More testing is therefore required.

## Conclusion

This review reveals the importance of support strategies when implementing EBP in community, including regular meetings and updates from the researcher, the allocation of resources and managerial support. This included training and time to become familiar with the innovation. Furthermore, training must be embedded in practice and individual adoption is often influenced by the nurses’ actual or perceived skill-set and personal relationship with the innovation. More testing of the identified strategies and facilitators is required. The review findings support the emerging consensus that implementation research reports should describe an evaluation of its process (Hulscher *et al*., 2003; Sales, 2013); utilize theory; and explore sustainability of implementation.

## Supplementary material

For supplementary material accompanying this paper visit https://doi.org/10.1017/S1463423618000488. click here to view supplementary material
