# Supplementary material for: Strategies, facilitators and barriers to implementation of evidence-based practice in community nursing: a systematic mixed-studies review and qualitative synthesis
Source: Prim Health Care Res Dev. 2018 Aug 2;20:e6. doi: 10.1017/S1463423618000488 (PMC6476399; doi:10.1017/S1463423618000488)
Supplement: Supplementary file 1 [file S1463423618000488sup001.docx]

Supplementary File: Summary of implementation strategies, facilitators and barriers in each of the included studies

| **`Authors and Year** | **Resign Design, Methods and Participants** | **Innovation/Type of Intervention** | **Implementation Strategies** | **Facilitators** | **Barriers** |
| --- | --- | --- | --- | --- | --- |
| Amacher, A. et al (2016) | Exploratory sequential design; qualitative interviews followed by questionnaire. Forty GPs, 12 HCNs and 4 PTs participated.  Post-implementation. | Falls Prevention Programme (FPP). | X | - All participants were satisfied with the organization and process of FPP. Good organisation of the project, helpful documents and well acceptable expenditure of time encouraged professionals to participate. - The programme benefits perceived by GPs and HCNs. | - Divergent opinions of the health professionals towards the aim of the FPP. GPs stated that the FPP should prevent first falls, whilst the HCNs thought it should prevent future falls. - Only a minority of GPs and HCNs were satisfied with the multidisciplinary setting. |
| Andrew, J. et al (2013) | Seven focus groups 25 community nurses.  Pre-post-implementation | The Dignity Care Pathway (DCP). | - A collaborative research approach. - Focus groups to gain “context-specific evidence - Training and manual. | - Supportive collaborative relationships - An experienced community nurse acted as a project champion. - Pre-existing trusting nurse-patient relations | - Practical issues relating to the research and implementation – time constraints and service demands. - Some nurses felt the very structured tool hindered the natural flow of conversation. |
| Annells, M. et al (2011) | Evaluation suveys (N=36) and 16 nurses participated in 3 focus groups.  Pre-post-implementation | Best practice mental health screening and referral clinical pathway for generalist community nursing. The pathway comprised screening and assessment and nurse interventions commensurate with generalist community nursing practice. | - Consultation with a panel of experts and focus groups. - Information session and plain language summary. - Education session held at a later date and further support was available. | - Rapport and trust between the nurses and clients contributed to successful use of the pathway. | - Participants were ‘time poor’. Pathway was too lengthy, unclear and not succinct. - Nurses felt inadequately skilled to undertake the additional work Organisational changes. |
| Brennan, et al (2010) | Surveys (60 nurses, 282 patients).  Post-implementation | Technology-enhanced practice (TEP) - usual care supplemented with a web-based resources (taken from the HeartCare website); system monitoring (e.g. symptom checklists, weight tracker, heart rate tracker); and communication (my goals, my journal, email, and bulletin boards). | - Training. - “First-visit support” and further training. - Monthly meetings with the research liaison nurses to provide project updates. - On-going troubleshooting. - Bi-monthly newsletter provided project update and motivational messages. | - Continued support from both internal and external facilitators. - The innovation was tailored to fit the nurses’ practice’ | - Nurses’ comfort with the technology. - Organizational changes and modifications in basic technology. - Difficulties using the innovation. |
| Bryon, S. & Hoskins, R. (2013) | Nine Semi-structured interviews (4 DNs and 5 community staff nurses).  Post-implementation | Verification of expected death (VOED) Policy. | - Mandatory training. | - Pre-existing trusting nurse-patient relations. - Regular updates in small-group formats, offering nurses the opportunity to share experiences. - Evidence that the intervention supported families facing bereavement. | - Expansion of the nursing role was considered inappropriate. - Inadequate training to deliver the innovation. - Fear and anxiety. |
| Doran et al (2013) | Interviews (n=12), 5 focus groups and surveys (n=118 at time 1, n=81 at time 2).  Post-implementation. | A clinical information system (CIS) in a community setting. Community nurses were provided access to clinical documentation, evidence-based practice resources through BlackBerry devices, e-mail, and a secure portal. They had access to two internet resources: the Registered Nurses Association of Ontario (RNAO) Best Practice Guidelines and the McMaster University Nursing Plus library. | - The CIS was designed collaboratively. - Training. - Management support and problem solving tools. | - Contributes to professional development and meets organisational goals. - Clear patient benefit and measurable outcomes. - Time to practise using the device. - Regular team meetings. | - Difficulties connecting clinical outcome improvements to the innovation Inadequate training - Independent nature and decentralised environment inhibited the CIS adaptation - Limited capabilities of the device - Time constraints. - Limited awareness of how or when to use research in daily practice. |
| Haycock-Stuart, E. & Kean (2013) | 29 nurses working in the community setting take part in 31 interviews (the nurse directors were interviewed twice, at the beginning and end of the data collection). Three focus groups  Post-implementation. | Policy to shift location of care from secondary, acute care settings to primary, community care settings. | - Top-down approach. | - ‘Bottom-up’ approach – ownership of the policy and the changes being implemented. - A “shared vision”. - The organisational infrastructure was adequately prepared, resourced and responsive enough | - ‘Top-down approach’ caused resistance. - Poor working relationships. - Nurses described being ill-prepared for the shift in the balance of care. - Nurses found themselves depleted of the organisational resources needed to fulfil the policy agenda, causing frustration. - Role conflict |
| Joy, H. et al (2015) | A collaborative project - assessment. The caseload comprised 37 patients. Pre- and post-implementation. | Foam dressing. | - Training. - Patient information, explaining the benefits, for use at visits. | - The adoption of the product saved nursing time. Time gained in reduced visit frequency meant more time could be spent with patients, increasing quality of visits and ensuring patients felt valued. - HCPs were willing and had the drive to adopt the product. - Comprehensive support, including staff meetings throughout, and education for change. - Early engagement, fully informed staff and clear definition of planned change. | - Difficult to gain clinical engagement when services are stretched due to increase demand. |
| Kapp, S. (2013) | 21 nurses completed the feedback survey and 218 client profiles were generated.  Pre-post-implementation. | Australian Wound Management Association (AWMA) Guidelines. | - Education session. - Face-to-face, telephone and email support. - Nurse consultant was employed. | - Innovation easy to use. - Support from the clinical nurse consultant. | - Nurses could not attend training due to staff shortages. |
| Murray et al (2011) | 23 interviews were undertaken in total (10 for case study 1; 5 for case study 2; and 8 for case study 3). Post-implementation | 1) Choose and Book (C&B) system in a hospital trust and the lead Primary Care Trust providing referrals to the hospital.  2) Picture Archive and Communication System (PACS) in one acute hospital trust. PACS was a system for digitizing images such as X-rays, scans, or photographs.  3) Community Nursing Information System (CNIS) for district nurses (DNs). The CNIS consisted of hand-held wireless enabled Personal Digital Assistant devices (iPAQs). | - Implementation group. - One-to-one training. - Emotional reassurance from trainers. | - Improves working relations. - Innovation easy to use - Innovation fitted well with existing skills set. - Advantages to using the innovation “won over” reluctant clinicians. - Meets organisational and personal goals. - Strong leadership and resources. - Top-down approach. | - Lack of confidence in the innovation. - Negative impact on working relations. - Nurses with low IT literacy were nervous about using CNIS. - Not perceived as part of their role. - Organisational change. |
| Nilsson et al (2010) | Case study of two DNs.  Pre-post-implementation | An electronic messaging program via computers and mobile phones with an internet connection. | - Information provided before use of the technological innovation. - Technical support during the implementation period. | - Innovation incompatible with working practices. - Technical problems gave them a sense of uncertainty. | - Innovation facilitated the district nurses’ work and saved time. - Positive impact upon nurse-patient relationship. |
| Nordmark, S., Zingmark, K., and Lindberg, I. (2016) | Registered adverse events and system failures; web-based survey (n=171); and interviews with staff (12 RNs, 9 DNs, and 5 HCOs).  Post-implementation | Discharge Planning Process (DPP) and information exchange through an electronic information system. | - A discharge coordinator was appointed. | - Collectively perceived value of DPP. - Meeting together over organizational boundaries generated a collective view of the DPP. - Staff were engaged and saw it as a legitimate part of their daily work. - Nurses became experts in DP, leading to a better continuity and safer timely exchanges of information. - Experienced nurses had a better understanding of when, what, how and with whom the information should be exchanged. | - No consensus on who performed the DPP and how it was performed. Difficulty to know the borders between the community regulations and healthcare regulations. - RNs saw the DPP as an extra work and prioritised other tasks. RNs wanted DNs, HCOs and physicians to step up and take responsibility. - Information exchange during the DPP depended on the nurses’ skills, beliefs, and knowledge. A lack of knowledge impeded the DPP. - Workload demands and insufficient time. - Limited understanding of each other’s roles and workload. |
| Papuay et al (2010) | Pretest-post-test design. Pretest (n=5999); Post-test (6 months after) (n=5894 patients); post-test (18 months after) (n=6097 patients). | The Belgian Guidelines for Prevention of Decubitus Ulcers (BGPDU). A patient and family education programme for pressure ucler prevention in an organisation for home care nursing. | - Leaflet for patients and informal caregivers were designed by a working group. - Training. | - Involving nurses in the data collection was advantageous. | X |
| Pare et al (2011) | A total of 150 patient records were analysed: 77 paper records (pre-implementation) and 73 SyMO records (pre-implementation). A total of 101 nurses (74% response rate) completed the questionnaire. A total of 57 semi-structured interviews were conducted with nurses. A total of 223 patients returned the questionnaire.  Pre-post-implementation | SyMO - software to plan and organise nursing activities in patients' homes | - All nurses were provided with a laptop computer and use of the SyMO system was mandatory, | - Easy to use with a clear and understandable structure. - Improved the quality of care. - Reduce nurses’ workload. - More control over the caring situation. | X |
| Sherman, H. et al (2016) | Sixteen healthcare centres (HCCs) were randomly selected from five geographic medical areas. The 75 years olds registered at these centres (SG n = 176, CG n = 262) filled in the questionnaire before and after the invention. Thirty-five DNs participated.  Post-implementation. | Preventive home visits (PHV). | - Training with supportive materials. - Continued support and regular visits from the researcher. | - Getting to know the DN made the patients feel safe. Older people also felt validated by the DNs and that the DNs were competent in their work. | - Insufficient training. - Activity-heavy organisation |
| Smith et al (2013) | Nurses were recruited from 3 localities Two focus groups were conducted within each locality, 6-8 weeks apart, to support phases 1 and 2 of the study. Participant numbers for each focus group ranged from 5 to 12 nurses.  Pre-implementation | Case management (defined as a package of care that covers a range of activities, rather than a single intervention) within community nursing practice. | - Action research principles. - A senior nurse who was ‘well-connected’ was recruited as the case manager. | - Clear need for innovation. - Saves money. - Trusting Nurse-patient relationship - Professional development. - Improving working relations. - Shared understanding and vision. - Nurses were confident that they had a key role to play in supporting the people the innovation is targeted at. | - Confusion and lack of clarify over how to implement the innovation. - No clear clinical benefit. - The local context affected the organisation of case management. |
| Tapper, L. et al (2012) | 26 home care nurses completed an online survey.  Post-implementatio. | Tablet computers distributed to 75 home care nurses. The devices contained nursing documents with access to evidence-based clinical practice guidelines. | - Handheld device was selected by a committee. - Devices were distributed to nurses before they were expected to use it so that they had time to explore the functionality. - Training and interactive activities to provide nurses with practical experience. | X | - Nurses’ age, experience, attitude, knowledge, perception and intention to use the technology affected the implementation process. - Buy-in and anxiety from nurses. - Technical problems and the functionality of the device. - Difficult connecting clinical outcome improvement. - Time consuming. |
| Taylor et al. (2015) | Case study of four community health services in England. 68 community nurses, with various roles, participated. 5 semi-clinical and 8 non-clinical staff participated. 3 GPs and 21 managers participated.  Post-implementation. | Telehealth to monitor patients with Chronic Obstructive Pulmonary Disease and Chronic Heart Failure. | - Local champions promoted telehealth and supported staff. - A local lead collaborator was recruited in each site. | - Technology was viewed positively by nurses with the knowledge of what is on offer. - Support from ‘local champions’ after initial training. - Early positive experiences and the sharing of success. - Substantiating experiential knowledge. - Productivity gains. - Dedicated external roles. - Reliable and flexible technology. - Dedicated resources. - A simple and standard referral process. | - Restructuring of the community nursing team. Other changes were seen as priority. - Telehealth was not always on the nurses’ radar and was considered a “fad”. - Concern of impact on workload. - Mixed evidence on the telehealth’s cost and clinical effectiveness. - Inadequate training and no one overseeing implementation. - Lack of trust or confidence in the telehealth. - Top-down approach. - Equipment limitations and outdated technology. - Unclear goals of use. |
| Vabo, G. et al (2016) | Action research project. Four focus groups and 2 interviews with 23 HCPs (nurses and assistant nurses).  Post-implementation. | Guideline for assessing individual needs and instructions for using the guideline, including nursing documentation routines and training; in the context of move to a common electronic health record. | - Project leader visited each site once a month. Between meetings, the ‘key nurse’ was responsible for monitoring the process. - Training. | - Clear and visible leadership. - Ownership and ‘feeling safe’ in changing situation. - Recognising the importance for clinical practice. - Including all members of staff and providing information for all. | - Competing interests and demands. Nurses reverted back to “old habits”. - Attitudes and culture of the organisation. - Lack of support and inadequate training. |
| van der Plas et al (2014) | 24 GPs completed a questionnaire, and 7 GPs, 5 DNs and 2 palliative care consultants attended one of two focus groups.  Post-implementation. | PaTz (Palliative Thuis Zorg - Palliative Care at Home): general practitioners and district nurses meet on a regular basis (every 2 months) to identify patients with palliative care needs and to discuss care for those patients. | - A PaTz group was formed. - The chairpersons of the PaTz groups were trained before implementation. - All participants were supported by their organisation to attend meetings. | - Direct positive effect on patient care. - The PaTz offered a safe environment, which offered emotional support. - Saved time. - Shared vision in, and clear aim of implementation meetings   Improving working relations. | - No follow up - GPs outnumbered district nurses. This made the active engagement in discussions for district nurses difficult. |
| Whittemore et al (2013) | 2 nurses were interviewed during the program implementation and at the end of the program. 2 community health workers (CHWs) were interviewed during the process of implementation and 1 CHW was interviewed at the completion of the project.  Post-implementation | A modified diabetes prevention program (mDPP) provided by homecare nurses to adults of public housing communities at-risk for type 2 diabetes. | - Participatory approach. - Training and selected reading. - Researcher met every two weeks with the nurses. - Home care nurses were hired Implement the program and a community health worker was hired to assist with recruitment. | - Experienced nurses utilised their contacts. - Nurses felt their contribution was important and valued. - New working relations. | - Poor working relations. - Competing demands and personal issues affected their ability to assist in program implementation. |
| Wilcox et al (2010) | 17 providers agreed to participate and complete the required training. 16 nurses agreed to participate and complete the required training. 11 of 12 providers and 15 of 16 nurses completed training evaluations.  Pre-implementation | The Heart Healthy and Ethnically Relevant (HHER) Lifestyle Program. | - Kick-off lunch. - CD-ROM training, | - Cost-effective. - Improve patient care. | - Organisational challenges. - Additional burden on time. - High staff turnover. |
